# Tendon mechanobiology in the context of tendon biofabrication

**DOI:** 10.3389/fbioe.2025.1560025

**Published:** 2025-08-28

**Authors:** Clemens Gögele, Girish Pattappa, Herbert Tempfer, Denitsa Docheva, Gundula Schulze-Tanzil

**Affiliations:** ^1^ Institute of Anatomy and Cell Biology, Paracelsus Medical University, Nuremberg, Germany; ^2^ Department of Musculoskeletal Tissue Regeneration, Orthopaedic Hospital König-Ludwig-Haus, University of Würzburg, Würzburg, Germany; ^3^ Institute of Tendon and Bone Regeneration, Paracelsus Medical University Salzburg, Salzburg, Austria; ^4^ Austrian Cluster for Tissue Regeneration, Vienna, Austria

**Keywords:** tendon, tenocytes, mechanostimulation, mechanosensation, mechanotransduction, mechanoresponse, cyclic stretch, biofabrication

## Abstract

Tendons are often affected by injuries or tendinopathies, resulting in serious and long-lasting impairments. The repair capacity is very low with a high risk of rerupture. Nevertheless, early, moderate and intermittent functional training adapted to the healing process has been shown to support tendon healing. The mechanosensitive tenocytes are responsible for extracellular matrix (ECM) synthesis, a process that is highly dependent on their specific and local mechanotopographical niche. The mechanical stimuli are triggered by the surrounding ECM that are then recognized by the cells via mechanosensation, transduced via activated intracellular molecular cascades to initiate the mechanoresponse, a process known as mechanotransduction. Hereby, the activation of calcium (Ca^2+^) dependent channels plays an essential role. Moreover, tenocyte primary cilium has been strongly suggested to participate in mechanosensation and -transduction. The cellular mechanoresponse results in processes such as ECM remodeling, collagen fiber alignment, cell proliferation and migration. Diverse approaches have been developed to recapitulate the natural mechanoenvironment and to optimize tenogenesis. It still remains difficult to identify the threshold parameters that determine optimal mechanical stimulation of tenocytes. The diverse effects of mechanical loading on tenocytes are not yet fully understood, as 2D and 3D experiments have not led to consistent conclusions. Further research is needed to fully address the mechanomics of each tendon cell population to gain a more comprehensive picture of cellular mechanoresponses and interdependencies within the tendon tissue that could help to explain possible feedback mechanisms for the regulation of the tendon ECM after mechanical loading. In turn, such efforts and subsequent achievements can help to outlining advanced therapeutic strategies and physiotherapy protocols for tendon health. Future developments in the field of mechanically assisted tendon reconstruction include 4D applications and direct *in situ* bioprinting.

## Native extracellular matrix of tendons

Tendons and ligaments are part of the connective tissues in the body that transmit the forces generated in the muscle to the bone. This is possible due to their abundant and specialized ECM composition (>90% of tissue volume), which predominantly consists of collagens (main component is type I collagen and small levels of type III, V, XI, XII and XIV collagens), small leucine-rich repeat proteoglycans (SLRPs) (e.g., decorin, biglycan, fibromodulin and lumican), large proteoglycans (e.g., versican and small amounts of aggrecan), glycoproteins (lubricin, tenascin C, tenomodulin), adhesion proteins (fibronectin and laminin) and elastic fibers (elastin, fibrillins 1 and 2) alongside the predominant component, water (Varma et al., 2016; Pajala et al., 2009; Smith et al., 2014; Sun et al., 2020; Izu et al., 2021; Ansorge et al., 2009; Yin et al., 2019; Leahy et al., 2023; Eekhoff et al., 2021; Dex et al., 2016; Docheva et al., 2005). Tendons have a hierarchical structure, composed of collagen fibrils, that are grouped in fibers and fiber bundles of different size resulting in the formation of subfascicles and fascicles. The latter and the whole tendon are surrounded by sheathes of loose connective tissue. These layers are called the endotendon that surrounds the subfascicles or peritenon, which encompasses the tendon fascicles and that all together form the interfascicular matrix (IFM), mediating fascicle gliding. A connective tissue sheet named the epitenon surrounds the complete tendon (Schulze-Tanzil et al., 2022; Thorpe et al., 2015; Kannus, 2000; Patel et al., 2021). Due to the parallel collagen fiber alignment in the tendon fascicles that are along to the longitudinal axis of the tissue (in the loading direction), tendons exhibit a high uniaxial mechanical strength (Durgam et al., 2020).

## Cellular components of tendons

The described ECM components are synthesized by resident cell types, which are highly specialized fibroblasts, called tenocytes, and other tendon-related cell types such as tendon stem/progenitor cells (TSPCs), myofibroblasts, dendritic cells (DCs), macrophages, as well as so called tenophages (Zhang and Wang, 2010; Szczodry et al., 2009; Lehner et al., 2019). Furthermore, adaptive T cells, endothelial cells and pericytes are also found in tendons (Schulze-Tanzil et al., 2022). Overall, the cellular content is very low in tendons (<10%) (Schneider and Docheva, 2017). The tenocytes as resident cells within the tendon fascicles are longitudinally orientated in rows, whereas those tenocytes in the sheathes formed by the endo-/peri- (IFM tenocytes) and epitenon are less strictly ordered. Within the IFM peri- and epitenon, diverse blood- and lymphatic vessel-derived cell types as well as nerve fibers are located (Zamboulis et al., 2024).

## Tendon enthesis

In the gradient attachment zone between tendon and bone, known as the enthesis, there are entheseal progenitor cells that differentiate into fibrochondrocytes in the fibrocartilaginous transition zone and osteoblasts in the bone attachment area (Chen et al., 2023). The enthesis is a mechano-adaptive interface structure that is essential for movement within the joints and provides a firm attachment between tendons and bone, allowing stability and stress reduction between the tissues (Killian, 2022). Moreover, the fibrocartilaginous enthesis is characterized by a spatial regulation of the mineralization front that is mediated by sclerostin (Sost) expression. Yambe et al., have reported fewer sclerostin and fibrochondrocytes in Scleraxis-deficient mice leading to impaired enthesial structure, while Sost knockout mice showed an elevated mineral density and stiffness of their Achilles tendon enthesis (Yambe et al., 2024). During tendon development, cells contain a mechanosensing organelle structure known as the primary cilium that plays an essential role in enthesis formation, via mainly regulation of the hedgehog signaling pathway, although depending upon its mechanical challenges, other pathways are also stimulated. Specifically, mechanical stimulation during *in vivo* mouse training (a motor-powered treadmill with 10 m/min on a 0° declined lane for 10, 20 or 30 min, 5 days per week) increased the release of transforming growth factor beta 1 (TGF-β1) and promoted tendon healing in the enthesis region via stimulation of TGF-β signaling by the primary cilium (Xiao et al., 2022). The mechanical *in vitro* stimulation (0%–3% strain; 1 Hz, for up to 24 h) shortened and disassembled the primary cilium of human tenocytes depending upon the TGF-β receptor (Rowson et al., 2018). Other cilium-associated signaling pathways participating in mechanotransduction remain unclear (Fang et al., 2020).

## Sensoring of mechanical stimuli by tenocytes

**FIGURE 1:**
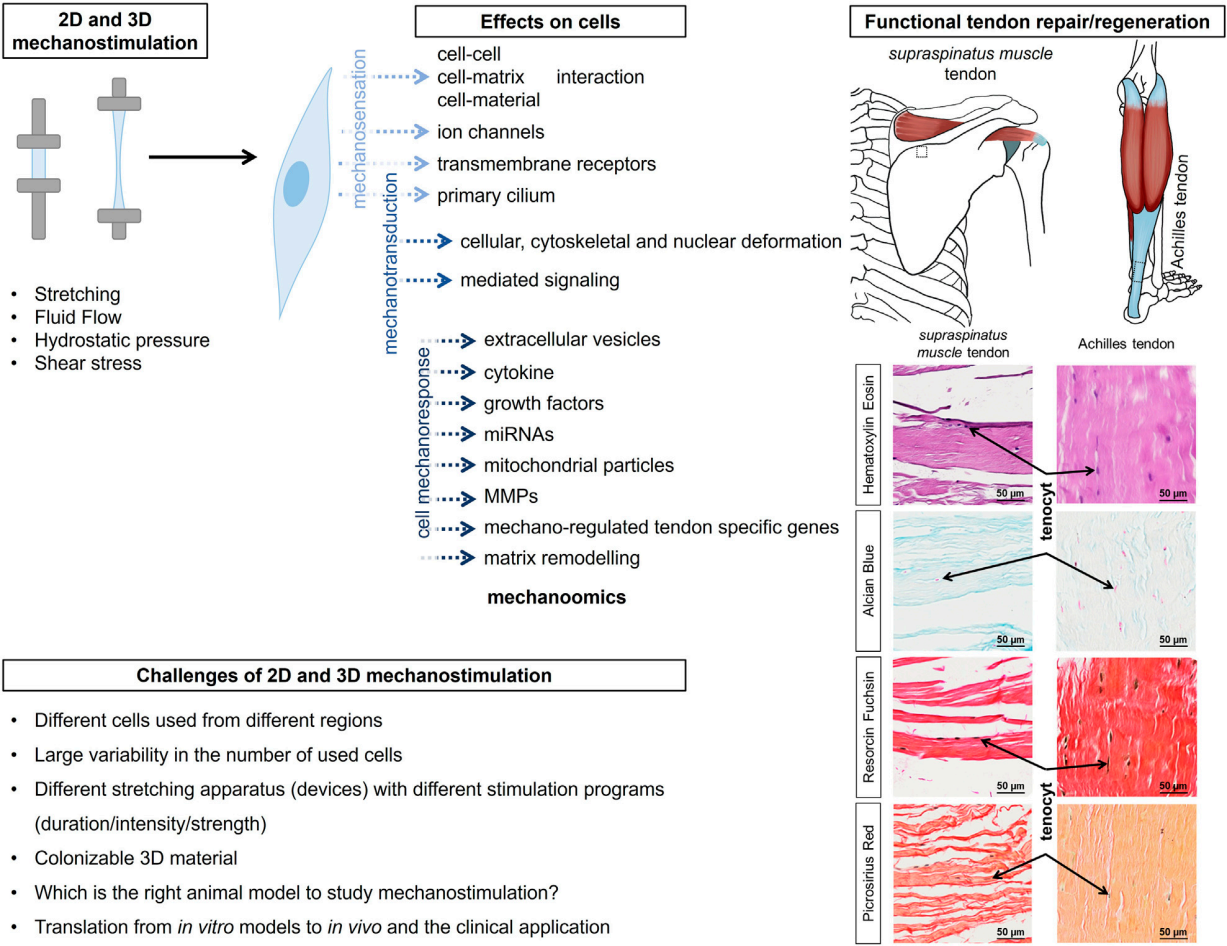
The graphical abstract summarizes the effect of 2D and 3D stimulation on tendon-resident cells for rapid and functional repair/regeneration of tendons, as shown here using the example of the supraspinatus muscle tendon and the Achilles tendon. The inserts show histologically images (Hematoxylin Eosin, Alcian blue, Resorcin Fuchsin and Picrosirius Red staining) and reveal tenocytes lined up like a string of pearls and embedded in or between the collagen fibre bundles. The histological images have been aligned so that they are similar to the anatomical images shown. MMP, matrix metalloproteinases; ECM, extracellular matrix; FAK, focal adhesion kinase; PI3K, phosphoinositide-3-kinase; Mkx, Mohawk; RhoA/Rock, Rho-associated protein Kinase/Rho-associated coiled-coil Kinase, TSPC, tendon stem/progenitor cells.

Tenocytes detect mechanical signals (e.g., movements of fibers, or unique biomechanical properties such as stiffness of their environmental substrates) with specific mechanosensitive channels and transmembrane receptors (e.g., connexins, integrins) within their cell membrane (Table 1; Table 2). Many of these receptors are located on the primary cilium (Khayyeri et al., 2015), providing cell surface enlargement and acting as mechanosensory non motile antennae (Lavagnino et al., 2011). As a typical mechanoreceptor of tenocytes, Passini et al., reported that the mechanosensitive ion channel PIEZO 1 underwent conformational changes and allowed a shear-stress induced calcium signaling during mechanical loading of tendons (Table 3) (Passini et al., 2021; Nakamichi et al., 2022). This Ca^2+^ influx is crucial for initiating downstream signaling pathways that govern tenocyte function and tendon remodeling (Magra et al., 2007). Furthermore, voltage-operated calcium channels (VOCCs), particularly L-type channels like CaV1.2 and TWIK related K^+^ (TREK)1 channels, are expressed in tenocytes and contribute to calcium signaling upon mechanical loading (Table 3). Activation of these channels enhances intracellular Ca^2+^ levels, influencing tenocyte behavior (Li H. et al., 2023; Magra et al., 2007). There are further mechanosensitive ion channels like the transient receptor potential (TRP) channels, which form a broad family of non-selective cation channels and especially TRPV4 can reduce mechanically induced Ca^2+^ signaling (Bian et al., 2024). The stretch-activated channels (SACs), which are also non-specific cation channels that open in response to membrane tension and initiate intracellular signaling cascades such as mitogen activated protein kinase/extracellular signal-regulated kinase (MAPK/ERK) and *Wingless* (Wnt) via Ca^2+^ (Millward-Sadler and Salter, 2004) (Table 4). Although not directly mechanosensitive, the P2X, especially P2X7 receptors are activated by ATP release during mechanical strain in tenocytes and contribute to inflammatory signaling and ECM remodeling (Yue et al., 2016) (Table1). This initial process of mechanical signal detection is called mechanosensation (Figure 1).

**TABLE 1:** The main role of different connexins to sense mechanical stimuli in tenocytes

Protein	Affected cell population (cell density and adhesion time)	Culture system/device	Stretch parameter and duration	Observations	References
Mechanosensation					
Transmembrane receptor					
Connexin 43	Rabbit Achilles tenocytes; (5 × 10^5^ per device; N.A.)	2D *in vitro* model, PDMS chambers; custom made;	Tensile strain with 0, 4 or 8% for 1, 2, 4, 6 or 24 h	4% static strain: intercellular communications ↑ via Cxn43 ↑ 8% static strain: intercellular communications ↓ Cxn43 gene expression↓	Maeda et al. (2017)
Connexin 43	Mouse model: supraspinatus and Achilles tendons; (N.A.)	*in vivo* model	10 m/min for 30 min followed 12 m/min per d for 2 days	Essential role for Cxn43 in postnatal tendon enthesis formation	Shen et al. (2020)
Connexin 43	Chicken flexor digitorum profundus avian tenocytes Human flexor digitorum profundus tenocytes, human flexor carpi radialis tenocytes, human flexor digitorum superficialis tenocytes; (5 × 10^5^/well) 5 days	2D *in vitro* model, (Flexcell™ International)	Cyclic strain of 3 or 5%; 1 Hz for 1, 6, or 12 h	5% (1 h): Cxn43 ↑ (both species)	Wall et al. (2007)

Abbreviations: Cxn, Connexin; d, days; h, hours; Hz, Hertz; m, meter; min, minutes; N.A.: not available; ↑: upregulation; ↓: downregulation.

**TABLE 2:** The main role of different transmembrane receptors to sense mechanical stimuli in tenocytes

Protein	Affected cell population (cell density and adhesion time)	Culture system/device	Stretch parameter and duration	Observations	References
Mechanosensation					
Transmembrane receptor					
Integrin alpha1	Human Achilles TSPC; (1 × 10^5^ cells/6 × 3 cm silicon dishes), 4 days	2D *in vitro* model (custom made device)	Stretched cyclically and uniaxially with a magnitude of 1%, 5% or 8%; 1 Hz continuous for 60 min (1 day) or intermittent on 3 consecutive d for 60 min/d	5%, 8% (3 days): ↑ integrin alpha 1	Popov et al. (2015)
Integrin alpha 11	Human Achilles tendon stem/progenitor cells (TSPC); (1 × 10^5^ cells/6 × 3 cm silicon dishes), 4 days	2D *in vitro* model (custom made device)	Stretched cyclically and uniaxially at with a magnitude of 1%, 5% or 8%; 1 Hz continuous for 60 min (1 day) or intermittent on 3 consecutive d for 60 min/d	TSPC showed ↑ integrin alpha 11 after cyclic stretch (1% after 1 day and 8% after 3 days)	Popov et al. (2015)
Integrin alpha 2	Human Achilles TSPC; (1 × 10^5^ cells/6 × 3 cm silicon dishes), 4 days	2D *in vitro* model (custom made device)	Stretched cyclically and uniaxially at with a magnitude of 1%, 5% or 8%; 1 Hz continuous for 60 min (1 day) or intermittent on 3 consecutive d for 60 min/d	TSPC showed ↑ integrin alpha 2 after cyclic stretch (1% after 1 day, 1% and 8% after 3 days	Popov et al. (2015)
Integrin beta 1	Human hamstring tenocyte; s (N.A)	2D *in vitro* model, (Flexcell™ International)	Uniaxial cyclic strain (0.1 Hz frequency, 10% strain) with 10 s rest for 1,000 cycles per d for up to 10 days	10% mechanical stimulation of integrin β1 leads to the phosphorylation of AKT	Mousavizadeh et al. (2020)
N-cadherin	Tenogenically differentiated hMSCs; (10^4^/cm^2^; 72 h)	2D *in vitro* model, elastic silicon chambers, (Strex Inc.)	Uniaxial cyclic strain for 6, 24, 48 and 72 h	Cyclic stretch (4%, 8% 12%) N-cadherin↑	Nam et al. (2019)

Abbreviations: d, days; h, hours; Hz, Hertz; hMSC, human mesenchymal stem cells; m, meter; min, minutes; s, seconds; TSPC, tendon stem/progenitor cells; N.A.: not available.

**TABLE 3:** The main role of different ion channels to sense mechanical stimuli in tenocytes

Protein	Affected cell population (cell density and adhesion time)	Culture system/device	Stretch parameter and duration	Observations	References
Mechanosensation					
Ion channel					
PIEZO1	Tail tendon derived tenocytes of skeletally mature female rats; (human: flexor digitorum, gracilis, semitendinosus, tenocytes); (3.8 × 10^4^ cells/cm^2^; N.A.)	*Ex vivo* tendon fascicles; 2D PDMS chamber; custom made	(Shear stress of around 0.01 Pa low, 0.01% strain per s; medium, 0.1% strain per s; high, 1.0% strain per s) from 0% to 10% strain; flow rate of 0.1 mL min^−1^)	PIEZO1 regulates the tissue stiffness by adjusting the collagen cross-linking	Passini et al. (2021)
Stretch activated calcium channel (SACC)	Tenogenically differentiated hMSCs; (1 × 10^5^; 72 h)	2D *in vitro* model, silicone chamber (Strex Inc.)	Cyclic loading with sinusoidal waveforms to 8% strain amplitude at a frequency of 1 Hz for 6, 24 48, and 72 h	8% strain: via SACC →hMSC tenogenic differentiation	Nam et al. (2017)
Tandem pore domain potassium channel (2 PK^+^) TREK-1	Human patellar tenocytes	N.A.	N.A.	Expression of mechanosensitive 2 PK^+^ channel TREK-1	Magra et al. (2007)
TRPV4	Fibrochondrocytes from Achilles tendon of male mice; (5 × 10^4^ cells per well; N.A.)	2D *in vitro* model (Nepa Gene Co., Ltd.)	Uniaxial cyclic stretch system 8%; 1 Hz; 8 h	Mechanical stimulation ↑ TRPV4 protein expression	Bian et al. (2024)
Voltage operated calcium channels (VOCCs) Ca _α1A_ Ca _α1C_ Ca _α1D_ Ca _α2δ1_ Ca _V1.2_	Human patellar tenocytes; mice Achilles tenocytes; (N.A.)	*In vivo* model N.A.	Uniaxial 1% strain/s	1% strain: ↑ Ca^2+^ signaling through Ca _V1.2_ growth factor myostatin↑ many ECM proteins for proliferation collagen fibrillogenesis↑	Magra et al. (2007) Li et al. (2023a)

Abbreviations: d, days; h, hours; Hz, Hertz; hMSC, human mesenchymal stem cells; m, meter; min, minutes; s, seconds; SACC, stretch activated calcium channel; TSPC, tendon stem/progenitor cells; PDMS, polydimethylsiloxane.

**TABLE 4:** Summarizes the activated key signaling pathways of tenocytes or tenogenic cells in 2D and 3D culture.

Activated signaling pathways	In 2D cultured tenocytes/MSCs	In 3D cultured tenocytes
Integrin-mediated Focal Adhesion Kinase (FAK)/ Phosphoinositide-3-kinase (PI3K)/AKT Pathway mTOR pathway FAK as an Upstream Regulator of PI3K/AKT FAK-Src Complex and Downstream Signaling	Mechanical loading activated integrins, leading to the recruitment of FAK. FAK subsequently activated the PI3K/AKT pathway, promoting tenogenic differentiation and cytoskeletal reorganization (Wang et al., 2018a) In fibroblasts, β1 integrin activation promoted phosphorylation of FAK, the p85 subunit of PI3K, and AKT. Inhibition of FAK attenuated PI3K and AKT phosphorylation, highlighting FAK’s role upstream in this signaling cascade (Xia et al., 2004). Integrin activation led to FAK autophosphorylation, creating a docking site for Src. The FAK-Src complex activated downstream molecules, including PI3K and AKT, contributing to cell survival and proliferation (Xia et al., 2004)	Activation of PI3K/AKT signaling pathway plays an essential role in tenogenic differentiation with 6% 0.25 Hz 8 h/d 6 days uniaxial mechanical loading (Wang et al., 2018b) Mechanical stretching activated the AKT/mTOR pathway via β1 integrin; AKT/mTOR then induced the phosphorylation of 4E-BP and S6 to regulate collagen expression in bioartificial tendons (Mousavizadeh et al., 2020)
Integrin α2β1/FAK/PI3K/AKT Pathway in Tenogenic Differentiation	Tendon stem/progenitor cells (TSPCs) cultured on micropatterned silk fibroin films showed α2β1 integrin mediated regulation through the FAK/PI3K/AKT signaling pathway, promoting tenogenic differentiation (Lu et al., 2023)	
Integrin-mediated RhoA/Rock Pathway	Downstream of integrin signaling, the RhoA/ROCK pathway regulated actin cytoskeleton dynamics and cell contractility, essential for tenocyte alignment and function (Xu et al., 2012; Melzer et al., 2021)	On rope like silk scaffolds, MSC showed tenogenic differentiation through the Rho/ROCK Pathway (Maharam et al., 2015)
Integrin-mediated MAPK Pathways (ERK1/2 and p38)	Mechanical stimulation induced activation of MAPK pathways, including ERK1/2 and p38, which were involved in tenocyte proliferation, migration, and expression of tendon-related genes (Cenni et al., 2024; Blache et al., 2021)	
TGF-β/Smad Signaling	Mechanical forces can activate TGF-β receptors, leading to Smad2/3 phosphorylation and nuclear translocation, which modulated gene expression related to ECM organization and tenocyte morphology (Maeda et al., 2011; Melzer et al., 2024)	Smad 2/3 pathway of tenogenic differentiated MSC is activated in the presence of cyclic tensile strain (Grier et al., 2017)
Hippo Pathway (YAP/TAZ)	Mechanical cues influenced the localization and activity of YAP and TAZ, transcriptional coactivators that regulated gene expression associated with cell proliferation and differentiation in response to substrate stiffness and mechanical stretch (stem cells and tenocytes) (Virdi and Pethe, 2021; Subramanian et al., 2018)	
Wnt/β-Catenin Signaling	MKX is stimulated by mechanical stretch as a mechanoregulated gene and is involved in tendon healing and protection of tendon damages by suppressing the Wnt/β-catenin pathway (Liu et al., 2024).	
Calmodulin-dependent Protein Kinase Kinase 2 ... 5′Adenosine Monophosphate-activated Protein Kinase (AMPK) Signaling)	Stretch-induced Ca^2+^ influx activated CaMKK2/AMPK signaling and FAK-cytoskeleton reorganization (Huang et al., 2022)	

Abbreviations: ERK, extracellular-signal regulated kinase; MAPK, Mitogen-activated protein kinase; TAZ, transcriptional co-activator with PDZ-binding motif; TGF, transforming growth factor; YAP, Yes-associated protein. ECM, extracellular matrix; FAK, focal adhesion kinase; PI3K, phosphoinositide-3-kinase; Mkx, Mohawk; RhoA/Rock, Rho-associated protein Kinase/Rho-associated coiled-coil Kinase, TSPC, tendon stem/progenitor cells.

## Mechanotransduction

Then, the mechanical impulse is transduced into biochemical signals leading to a cell response - a process called mechanotransduction. In more detail, van Helvert et al., defined mechanotransduction as a cellular response to tissue organization (ECM stiffness, confinement and topology) and mechanics at subcellular, cellular and multicellular levels generated by the multifaceted interactions between the plasma membrane of the cells and the substrate (van Helvert et al., 2018). Mechanotransduction depends on the interplay between calcium ions (Ca^2+^) and tenocytes. Mechanosensitive ion channels in tenocytes mediate an influx of Ca^2+^ in response to mechanical forces such as tensile strain or fluid flow shear stress. This Ca^2+^ influx activates signaling cascades, which regulate various cellular processes, including cytoskeletal organization, gene expression, and ECM production (Maeda et al., 2013; Huisman et al., 2014). Simultaneous application of cyclic tensile strain and fluid flow shear stress results in a more pronounced increase in intracellular Ca^2+^ concentration compared to each individual stimulus. This synergistic effect underscores the complexity of mechanical environments *in vivo* and their impact on tenocyte physiology (Maeda et al., 2013). Furthermore, mechanical stimulation can lead to the propagation of Ca^2+^ waves between tenocytes via gap junctions. Mechanical stimulation can produce a Ca^2+^ wave that propagates via the passage of inositol 1,4,5-trisphosphate (IP_3_) through gap junctions which interconnect neighboring tenocytes. IP_3_ then acts upon IP_3_ receptors in the endoplasmic reticulum (ER) to release intracellular Ca^2+^ into the cytosol. This mechanism highlights the role of gap junctions in facilitating intercellular calcium signaling in response to mechanical stimuli in tenocytes (Lavagnino et al., 2015). Mechanical stimulation of a single cell can initiate intercellular Ca^2+^ waves that propagate to the tendon tissue network of tenocytes interconnected by gap junctions. These findings suggest that gap junctions play a crucial role in the transmission of Ca^2+^ signals between cells in response to mechanical stimuli (Iyyathurai et al., 2016; Wall and Banes, 2005). This intercellular communication amplifies the mechanotransduction signal, coordinating a collective cellular response to mechanical cues.

The extracellular Ca^2+^ ion concentration ([Ca^2+^]_e_) modulates tenocyte responsiveness to mechanical stimuli. Alterations in [Ca^2+^]_e_ can affect the gating of mechanosensitive channels, thereby influencing the magnitude of intracellular Ca^2+^ signaling and subsequent cellular responses (Wall and Banes, 2005; Chen W. et al., 2015).

Mechanical impulses, registered by integrins as transmembrane receptors, activate downstream signaling cascades, like focal adhesion kinase (FAK), which subsequently trigger the MAPK/ERK and phosphoinositide-3-kinase (PI3K/AKT) pathway. These pathways are crucial for tenocyte migration, proliferation, survival and ECM synthesis (Stanczak et al., 2024). The Yes-associated protein (YAP) and transcriptional coactivator with PDZ-binding motif (TAZ) cascade is a further signaling pathway, which is turned on by mechanical stimulation and influences gene expression related to cell proliferation and ECM production in tendon cells (Randelli et al., 2020) (Figure 1). Mechanical overloading activated the mechanistic target of rapamycin (mTOR) pathway, which can lead to non-tenocyte differentiation and contribute to tendinopathy. Inhibition of mTOR with rapamycin has been shown to mitigate these effects (Nie et al., 2021). An overview of activated key signaling pathways after mechanostimulation was summarized in Table 4.

## Effect of mechanical stimuli on cytoskeleton, tendon-specific matrix and cytokines

The mechanotransduction led to nuclear deformation and influenced in the next step the depolymerization of filamentous actin fibers in rat Achilles tenocytes (Chen et al., 2018; Xu P. et al., 2021; Lammerding et al., 2004; Pancheri et al., 2025) (Table 5). This also resulted in a complete reorientation of the cellular structure. Depending on the degree of stretching and strength, this can have different effects on the cytoskeleton (Gögele et al., 2021a; Fleischmann et al., 2024) (Table 6).

**TABLE 5:** Reports on the effect of mechanotransduction on the nuclear deformation.

Protein	Affected cell population (cell density and adhesion time)	Culture system/device	Stretch parameter and duration	Observations	References
Mechanotransduction					
Nuclear deformation					
Chromatin Remodeling	Tenocytes; 1 × 10^5^ cells (2 × 2 cm), overnight	2D *in vitro* model, cyclic stretching device (Strex Inc.)	10%; 0.5 Hz; 1 h	Uniaxial stretch actin stress ↑ fiber formation, ↑ chromatin decondensation and ↑ regulated tenomodulin gene expression, which might then regulate tenocyte migration.	Xu et al. (2021b)
Lamin A/C (LMNA)	Mouse embryonic fibroblasts; (900 cells/cm^2^; N.A.)	2D *in vitro* model	17.4%–19.8% membrane strain	↑ nuclear deformation, defective mechanotransduction and ↑ viability in LMNA^−/−^	Lammerding et al. (2004)
Linker of Nucleoskeleton and Cytoskeleton (LINC) Complex (Nesprins and SUN Proteins)	Mouse Achilles and tail tendons	3D *in vitro* model	10 cycles of 1%/s	Nuclear mechanotransduction through LINC played a role in regulating tendon formation during neonatal development	Pancheri et al. (2025)

Abbreviations: LINC, linker of nucleoskeleton and cytoskeleton; LMNA, lamin.

**TABLE 6:** Effect of mechanostimulation on secretion of, growth factors, tendon related transcription factors and cytokines.

Protein	Affected cell population (cell density and adhesion time)	Culture system/device	Stretch parameter and duration	Observations	References
Cell response					
F-Actin	Rat Achilles tenocytes; (4 × 10^4^ cells (N.A.), 12 h; (15% tension, 1 Hz for 24 h) Rat Achilles tenocytes; (1,25 × 10^4^ cells/cm^2^ in PDMS chambers after 48 h adhesion time)	2D *in vitro* model, custom made device	15%; 1 Hz; 24 h Uniaxial cyclic stretch (14%; 0.3 Hz; 48 h)	Stretching induced the depolymerization of F-actin and fiber alignment in direction of zero stretch	Chen et al. (2018)

Abbreviations: d, days; h, hours; Hz, Hertz; PDMS, polydimethylsiloxane.

Moreover, cell-cell communication between the tenocytes within the longitudinal rows, as well as between the rows, via their plasmalemma extensions passing through the abundant ECM plays a pivotal role to synchronize mechanoresponse via gap junction connections (mainly composed of connexins 32 and 43) at the tissue level (Waggett et al., 2006). There also seem to exist multiple other means of cell-cell communication, facilitating the cell reaction to mechanical stimuli in tendons. Mediators can be released by extracellular vesicles (EVs) in tendon (Song et al., 2023; Zhu et al., 2022). Proliferation, migration and tenogenic differentiation of TSPCs can be induced by small EVs and their local application could be seen as a novel approach to treat tendon injuries (Zhang et al., 2023). It was shown, that rat TSPCs express mechanosensitive miRNA-337-3p upon mechanical loading and the overexpression of this miRNA could effectively rescue *in vivo* ectopic ossification in rat tendinopathy (Geng et al., 2020). Egerbacher et al., postulated that tenocyte intercellular communication can also occur via ultrathin intercellular structures, called nanotubes (Egerbacher et al., 2020; Waggett et al., 2006). In addition, cell-cell communication in tendons in response to strain-stress can obviously take place through extracellular mitochondrial particles (ExtraMito) from fragmented mitochondria, that are released into the ECM and can regulate the immune microenvironment and recruit macrophages after mechanical stimulation (Chen et al., 2024).

Furthermore, mechanostimulation evokes a release of growth factors including basic fibroblast growth factor (bFGF)2, Early growth response (EGR)1 and 2, Insulin-like growth factor 1 (Scott et al., 2007; Skutek et al., 2001; Herchenhan et al., 2020; Lejard et al., 2011). However, not all of them were upregulated after mechanical stimulation, as TGF-β3 has been shown to be reduced after cyclic stretch (Legerlotz et al., 2013) (Table 7).

**TABLE 7:** Effect of mechanostimulation on secretion of growth factors.

Protein	Affected cell population (cell density and adhesion time)	Culture system/device	Stretch parameter and duration	Observations	References
Cell response					
Basic Fibroblast Growth Factor 2	Human patellar tenocytes; (1.5 × 10^5^ cells into silicon dishes (no specification how many dishers were used, 72 h)	2D *in vitro* model, silicon dish (6 × 3 cm cell culture surface)	Cyclic biaxial with 1 Hz; 5%; 15 and 60 min, experiments were concluded 2, 4 and 8 h after the end of mechanical stretching	↑ secretion of bFGF after 2 h	Skutek et al. (2001)
Early Growth Response 1 (EGR1)	Human semitendinosus and gracilis tenocytes; (2.5 × 10^5^ cells/well; N.A.)	2D *in vitro* model	Cyclic uniaxial mechanical stretch (sinusoidal strain, 2.5%; 1 Hz; 5 min)	↑ EGR1 (10% strain and not 2% strain) and peaked after 1–2 h, rapid response to overload	Herchenhan et al. (2020)
Early Growth Response 2 (EGR2)	Mouse flexor digitorum longus tendon	3D *in vivo* native samples from E11.5, E12.5 and E14.5	N.A.	The DNA binding proteins EGRs ↑ of collagen type 1A1,3A1, 5A1, 12A1, 14A1 expression during tendon cell differentiation in embryonic limbs	Lejard et al. (2011)
Insulin-like Growth Factor 1 (IGF1)	Rat supraspinatus tendons	3D *in vivo* tendon loading model	N.A.	↑ IGF-1 and therefore an altered tendon morphology	Scott et al. (2007)
Platelet-derived Growth Factor (PDGF)	Human patellar tenocytes; (1.5 × 10^5^ cells into silicon dishes (no specification how many dishes were used; 72 h)	2D *in vitro* model, silicon dish (6 × 3 cm cell culture surface)	Cyclic biaxial stretch with 5%; 1 Hz; 15 and 60 min, experiments were stopped 2, 4 and 8 h after the end of mechanical stretching	↓secretion of PDGF after 1 h, ↑ secretion after 2, 4, and 8 h	Skutek et al. (2001)
Transforming Growth Factor TGF-β1 TGF-β2 TGF-β3	Bovine common digital extensor tendon fascicles; (N.A.)	3D *in vitro* model	30% strain for 1 h	↓ TGF-β1, TGF-β2, TGF-β3	Legerlotz et al. (2013)
TGF-β	Human patellar tenocytes; (1.5 × 10^5^ cells into silicon dishes (no specification how many dishes were used; 72 h)	2D *in vitro* model, silicon dish (6 × 3 cm cell culture surface)	Cyclic biaxial with 5%; 1 Hz; 15 and 60 min, experiments were concluded 2, 4 and 8 h after the end of mechanical stretching	↑ secretion of TGF-β after 15 min and 1 h	Skutek et al. (2001)

Abbreviations: d, days; h, hours; Hz, Hertz; EGR, early growth response; FGF, fibroblast growth factor; IGF, insulin like growth factor; TGF, Transforming Growth Factor; N.A., not available.

Not only growth factors but also tenogenic transcription factors were upregulated after mechanical loading. In tendon, the mechanical stimulation leads to increased activation of the tenogenic mechanosensitive transcription factor, the Scleraxis basic helix loop helix transcription factor [Scx] (Table 8) (Gumucio et al., 2020; Papalamprou et al., 2023; Kubo et al., 2020), Mohawk [MKX] (Table 9) (Suzuki et al., 2016; Kayama et al., 2016; Liu et al., 2024), EGR1 (Herchenhan et al., 2020), EGR2 (Lejard et al., 2011) and other transcription factors including Runt-related transcription factor 2 [Runx2], sex-determining region Y-box 9 [Sox9]) (Table 10). In addition, mechanical stimulation induced an altered ECM synthesis in tenocytes including tendon-related glycoproteins such as tenomodulin (Dex et al., 2017; Xu P. et al., 2021) (Table 10). Through optimal adaptation to mechanical challenges, a remodeling process can take place (Xu K. et al., 2021; Popov et al., 2015). Maturation and aging trigger tenocytes to shift towards senescence and develop a senescence-associated secretory phenotype (SASP) by activation of the Janus kinase signal transducer and activator of transcription (JAK/STAT) pathway (Tinguely et al., 2023; Yan et al., 2020; Russo et al., 2015; Schulze-Tanzil et al., 2022), leading to dramatic changes in their mechanosensing and mechanotransduction capacity (Zuskov et al., 2020; McCrum et al., 2018; Epro et al., 2017).

**TABLE 8:** Effect of mechanostimulation on secretion of Scleraxis transcription factor.

Protein	Affected cell population (cell density and adhesion time)	Culture system/device	Stretch parameter and duration	Observations	References
Cell response					
Early tendon related transcription factors					
basic helix-loop-helix transcription factor (Scleraxis, Scx)	Mouse tendon; (N.A.)	3D *ex vivo* model	14 days after surgery	Tenocyte proliferation and ECM synthesis, critical transcription factor for tendon growth	Gumucio et al. (2020)
Scleraxis	Human induced pluripotent stem cells (iPSCs); (2 × 10^4^ cells/cm^2^)	N.A.	Sinusoidal strain of 4%; 0.5 Hz; 2 h/d; 7 days in total	Scx overexpression in iPSCs combined with stretching resulted in collagen-1 fiber alignment and Tnmd deposition	Papalamprou et al. (2023)
Scleraxis	Mouse Achilles tenocytes; (2.5 × 10^4^ cells/cm^2^ 24–36 h)	2D *in vitro* model, PDMS chambers	5%; 1 Hz; 3 h stretch	↑ after 1 and 2 Hz stretch	Kubo et al. (2020)
Scleraxis	Rat Achilles tenocytes; (N.A.)	3D *ex vivo* model	1-mm (3%; 0.5 Hz) and 2-mm (5%; 0.5 Hz)	↑ after 1-mm elongation	Saito et al. (2022)
Scleraxis	Equine superficial digital flexor tenocytes; (2 × 10^5^ cells)	Flexibles silicone culture plate (Flexcell™ International)	(1%; 0.5 Hz; 2 h) every 24 h for 3 days	Scx is required for the development of force-transmitting tendon during development and for mechanically stimulated tenogenesis	Nichols et al. (2018)
Scleraxis	Mouse Achilles and patellar tenocytes; (N.A.)	3D *in vitro* constructs	Uniaxial 3D stretching of 0%, 3%, 6%, or 9%; 8 h/d, followed by 16 h of rest	↑ Scx after 6% and 9% strain	Chen et al. (2024)

Abbreviations: d, days; h, hours; Hz, Hertz; iPSCs, induced pluripotent stem cells; Scx, Scleraxis; Tnmd, Tenomodulin; N.A., not available; PDMS, Polydimethylsiloxane; Scx, Scleraxis; ECM, extracellular matrix.

**TABLE 9:** Effect of mechanostimulation on secretion of Mohawk tendon related transcription factor.

Protein	Affected cell population (cell density and adhesion time)	Culture system/device	Stretch parameter and duration	Observations	References
Cell response					
Tendon related transcription factors					
Mohawk (MKX)	Mouse patellar tenocytes (2 × 10^5^ cells/10 cm^2^ elastic silicon rubber chamber, 12 h)	2D *in vitro* model with a uniaxial materials testing system (Strex Inc.)	Monoaxial cyclic elongation of 4%; 6 h	Accelerated tendon differentiation, orchestrated tendon cell maintenance and preventing chondrogenic/osteogenic differentiation (MKX^+/+^ tendon derived cells showed ↑ levels of tendon-related genes, such as MKX, Col1a1, and Col3a1, indicating the tenogenic differentiation, the same mechanical stimulation of MKX^−/−^ tendon-derived cells increased chondrogenic markers, such as SRY-box (Sox)6, Sox9 and Aggrecan, rather than tendon-related genes	Suzuki et al. (2016)
MKX	Rat Achilles tenocytes (N.A.)	2D *in vitro* model, (Flexcell™ International)	(0.5, 1, 2, 4, 8, and 10%; 0.25, 0.5, 1, and 2 Hz) 2% and 0.25 Hz for 6 h stretching	Mechanical forces regulated MKX expression	Kayama et al. (2016)
MKX	Mouse Achilles tenocytes (N.A.)	2D *in vitro* model, (Flexcell™ International)	Various stretch magnitudes (1%, 2%, 4%, 8% and 12%); 1.0 Hz; 4 h	MKX may be stimulated by mechanical stretch as a mechanoregulated gene, binding to Wnt3a or Wnt5a to repress osteogenesis and calcification in tendon tissues. Yet, excessive stimulus will break this balance to induce tendon damage and tendinopathy.	Liu et al. (2024)
MKX	Mouse Achilles and patellar tenocytes; (N.A.)	3D *in vitro* model	Uniaxial 3D stretching of 0%, 3%, 6%, or 9% strain for 8 h/d, followed by 16 h of rest	↑ MKX after 6% and 9% strain	Chen et al. (2024)

Abbreviations: d, days; h, hours; Hz, Hertz; MKX, Mohawk; N.A., not available; Col, Collagen; SOX, SRY box.

**TABLE 10:** Effect of mechanostimulation on secretion of other factors.

Protein	Affected cell population (cell density and adhesion time)	Culture system/device	Stretch parameter and duration	Observations	References
Cell response					
Late tendon related transcription factors					
Runt-related transcription factor 2 [Runx2]	Rat Achilles tenocytes, (N.A.)	3D *ex vivo* model	1-mm (3%; 0.5 Hz) and 2-mm (5%; 0.5 Hz)	↑ after 2-mm elongation	Saito et al. (2022)
Sex-determining region Y-box 9 [Sox9]	Rat Achilles tenocytes; (N.A.)	3D *ex vivo* model	1-mm (3%; 0.5 Hz,) and 2-mm (5%; 0.5 Hz)	↑ after 1-mm elongation	Saito et al. (2022)
Tenomodulin (TNMD)	Mouse Achilles and patellar tenocytes (N.A.)	3D *in vitro* constructs	Uniaxial 3D stretching of 0%, 3%, 6%, or 9%; 8 h/d, followed by 16 h of rest	↑ TNMD after 6% and 9% strain	Chen et al. (2024)
TNMD	Rat Achilles tenocytes; (1 × 10^5^ cells, 1 h)	2D *in vitro* model, (Strex Inc.)	Uniaxial cyclic stretching (10%; 0.5 Hz; 1 h)	↑ Tnmd	Xu et al. (2021b)
TNMD	Human Achilles tendon stem progenitor cells; (1 × 10^5^ cells) mouse TNMD knockout model	2D *in vitro* model: FBS-coated silicone dish	Uniaxial cyclic stretch (5%; 1 Hz; 1 h)	↑ TNMD under 5% cyclic strain. TNMD −/− model had ↓ running capacity	Dex et al. (2017)

Abbreviations: d, days; h, hours; Hz, Hertz; FBS, fetal bovine serum; SOX, sex determining region Y box, TNMD, tenomodulin.

**FIGURE 2:**
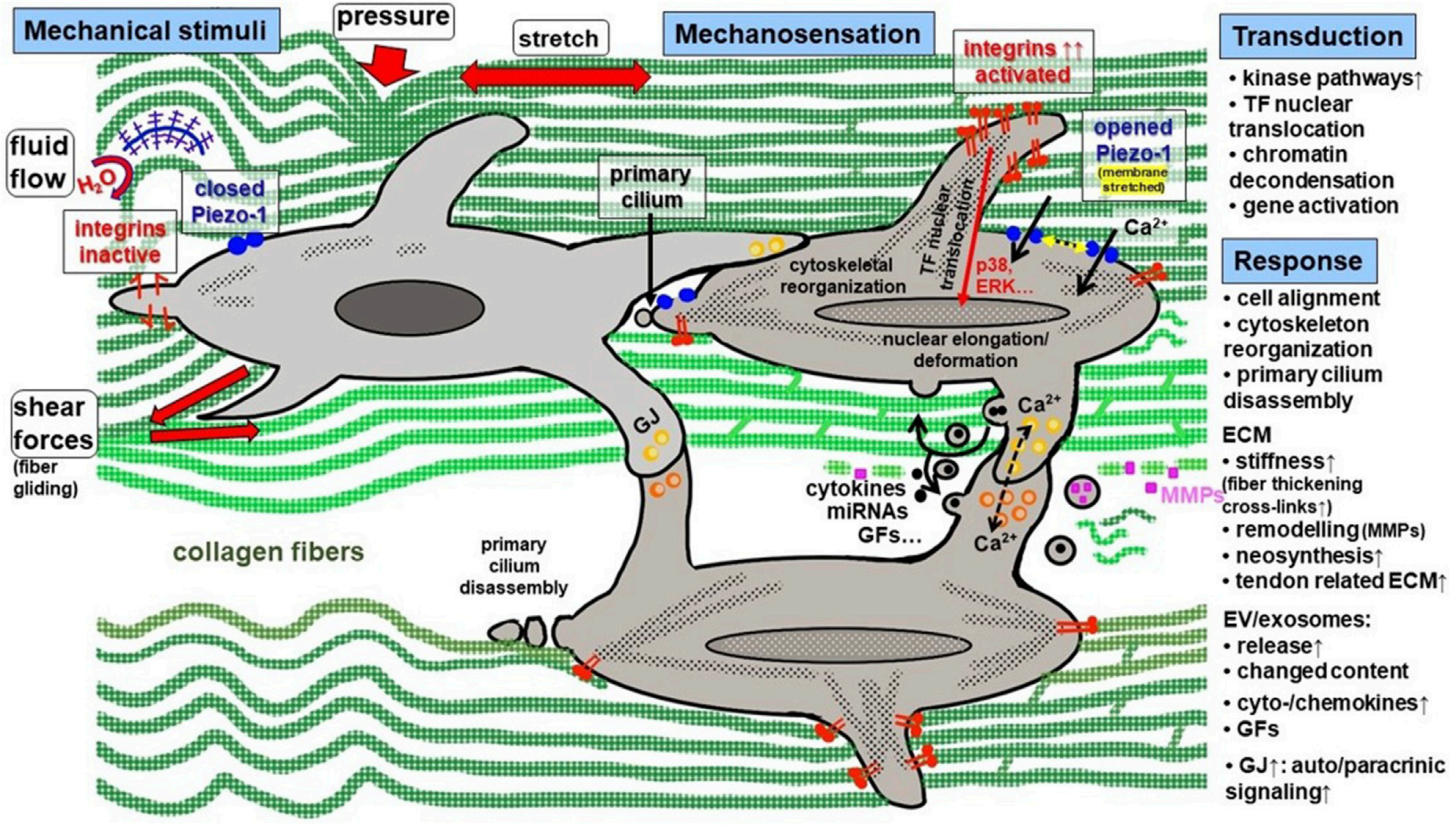
Synopsis of mechanical stimuli and the key steps of mechanosensation, -transduction and–response in tendon. Mechanical stimuli in tendon are: cyclic tension, pressure, fluid flow, shear forces. Mechanosensing is mediated by a group of receptors among them integrins (activated by changes in ECM stiffness) and Piezo-1 channels (activated by membrane stretching). In addition, the primary cilium is a mechanosensing organelle in tenocytes. It goes along with nucleus deformation. Mechanotransduction occurs in response to integrin activation, clustering, focal adhesion component activation, cytoskeleton reorganization and Ca^2+^ influx, leading to transcription factor nuclear translocation mediated by different activated pathways including typical kinases. Cell response comprises of the release of diverse factors and initiation of auto-/paracrinic response and neomatrix synthesis. Moreover, cells are synchronized by enhanced gap junction expression. ECM, extracellular tendon matrix; ERK, extracellular signal regulated kinase; EV, extracellular vesicles; GF, growth factor; GJ, gap junction; MMP, matrix metalloproteinase; TF, transcription factor.

The dynamic response of tendons to physical activities is enabled by their biomechanical visco-elastic behavior that is mediated via their unique ECM composition (Notermans et al., 2019). In addition to cyclic stretching, mechanical impulses on tendon cells evoke also repetitive fluid flow of water by binding to and releasing from negatively charged glycosaminoglycans, as well as shear forces caused by fascicle and fiber gliding (Mlyniec et al., 2021) and in some areas, pressure (Figure 2).

The remodeling of the ECM is accomplished by matrix metalloproteinases (MMPs), among other things. Mechanical stress has a significant influence on this remodeling through the increased or suppressed release of MMPs, as the following examples show. There was a decrease in MMP1 (Yang et al., 2005) and increase in other types (MMP9, 13, 14) (Saito et al., 2022; Nichols et al., 2018; Lohberger et al., 2016). Patel et al. postulated the upregulation of MMP3 after cyclic loading in a new fiber composite hydrogel may be early responses to changes in the tenocyte environment (Patel et al., 2018) (Table 11).

**TABLE 11:** Effect of mechanostimulation on secretion of mitochondrial particles and MMPs.

Protein	Affected cell population (cell density and adhesion time)	Culture system/device	Stretch parameter and duration	Observations	References
Cell response					
Mitochondrial particles	Mouse Achilles and patellar tenocytes; (N.A.)	3D *in vitro* constructs (custom made)	Uniaxial 3D stretching 0%, 3%, 6%, or 9%; 8 h/d, followed by 16 h of rest	Release of mitochondrial particles	Chen et al. (2024)
Matrix metalloproteinases [MMP] 1	Human rotator cuff tenocytes; (1 × 10^5^ cells/well; N.A.)	2D *in vitro* model (Flexcell™ International)	10%; 0.5 Hz; 7 and 14 days. Each cycle consisted of 10-s strain and 30-s relaxation	↑ MMP1 (after 7 and 14 days)	Lohberger et al. (2016)
MMP1	Human patellar tenocytes; (2 × 10^5^ cells in 6 × 3 cm silicon dish, 12–16 h)	2D *in vitro* model	4% or 8%; 0.5 Hz; 4 h	↓ MMP1 after 4%; ↑ MMP1 after 8%	Yang et al. (2005)
MMP3	Human primary rotator cuff Tenocytes; (1 × 10^5^ cells/well; N.A.)	2D *in vitro* model, (Flexcell™ International)	10%; 0.5 Hz; 7 and 14 days. Each cycle consisted of 10-s strain and 30-s relaxation	↑ MMP3 (after 7 and 14 days)	Lohberger et al. (2016)
MMP9	Porcine infraspinatus tenocytes; (2.5 × 10^4^ cells/cm^2^; 24 h)	2D *in vitro* model, (Strex Inc.)	10%; 0.5 Hz; 24 h	↓ MMP9	Hatta et al. (2013)
MMP9	Human Achilles TSPC; (1 × 10^5^ cells/6 × 3 cm silicon dishes), 4 days adherence	2D *in vitro* model (custom made device)	Cyclically stretched for 1%, 5% or 8%; 1 Hz; 60 min (1 day) or intermittent on 3 consecutive d for 60 min/d (3 days)	↑ MMP9 (5% after 3 days)	Popov et al. (2015)
MMP13	Rat Achilles tenocytes; (N.A)	3D *ex vivo* model model	1-mm (3%; 0.5 Hz,) and 2-mm (5%; 0.5 Hz)	↑ after 2-mm elongation	Saito et al. (2022)
MMP13	Human Achilles TSPC; (1 × 10^5^ cells/6 × 3 cm silicon dishes), 4 days adherence	2D *in vitro* model (custom made)	Cyclically stretched for 1%, 5% or 8%; 1 Hz; 60 min (1 day) or intermittent on 3 consecutive d for 60 min/d (3 days)	↑ MMP13 (1%, 5% and 8% after 3 days)	Popov et al. (2015)
MMP13	Human rotator cuff tenocytes; (1 × 10^5^ cells/well; N.A.)	2D *in vitro* model (Flexcell™ International)	10%; 0.5 Hz; 7 and 14 days. Each cycle consisted of 10-s strain and 30-s relaxation	↑ MMP13 (after 7 and 14 days)	Lohberger et al. (2016)
MMP14	Human rotator cuff tenocytes; (1 × 10^5^ cells/well; N.A.)	2D *in vitro* model (Flexcell™ International)	10%; 0.5 Hz; 7 and 14 days. Each cycle consisted of 10-s strain and 30-s relaxation	↑ MMP14 (after 7 and 14 days)	Lohberger et al. (2016)
MMP14	Human Achilles TSPC; (1 × 10^5^ cells/6 × 3 cm silicon dishes), 4 days adherence	2D *in vitro* model (custom made device)	Cyclically stretched for 1%, 5% or 8%; 1 Hz; 60 min (1 day) or intermittent on 3 consecutive d for 60 min/d (3 days)	↑ MMP14 (1%, 5% and 8% after 3 days)	Popov et al. (2015)

Abbreviations: d, days; h, hours; Hz, Hertz; MMP, matrix metalloproteinase; N.A., not available.

This cell response to mechanosensation orchestrates the release of mediators and the auto-/paracrinic response to them, including not only growth factors, but also cytokines, and neurotransmitters (Tsuzaki et al., 2003; Mousavizadeh et al., 2024; Skutek et al., 2001). Cytokines like interleukin (IL)-1β may act as a defense/survival factor in early cellular responses to mechanical loading (Qi et al., 2006). However, the release of IL-1β also depends strongly on the environment (2D or 3D) and the strain parameters (Yang et al., 2005). Especially, IL-6, that was already significantly upregulated after 1 h and 30% strain in native bovine foot extensor tendons and seemed to play a role in tendon adaptation by regulating healing processes (Legerlotz et al., 2013). This upregulation of IL-6 after cyclic loading could be seen too in a new fiber composite hydrogel (Patel et al., 2018). Skutek et al. investigated the effects of cyclic biaxial mechanical stretching (1 Hz frequency and 5% amplitude) on human tendon fibroblasts and found that such mechanical stimulation modulates the secretion patterns of various growth factors, and the cytokine IL-8. Their findings suggest that mechanical stretch can indeed induce IL-8 expression in these cells (Skutek et al., 2001). Mechanical stretching of human patellar tendon fibroblasts controlled the expression of pro-inflammatory markers. To date, however, there is no uniform opinion regarding the connection between increased stretching and inflammatory markers. Specifically, low-magnitude stretching (4%) reduced IL-1β-induced expression of MMP-1, cyclooxygenase-2 (COX-2), and prostaglandin E2 (PGE2) production, whereas high-magnitude stretching (8%) enhanced their expression. Furthermore, cyclic mechanical stretching of human tendon fibroblasts increased PGE_2_ production in a magnitude-dependent manner. Stretching at 8% and 12% of silicon membranes for 24 h resulted in 1.7-fold and 2.2-fold increases in PGE_2_ production, respectively, compared to non-stretched controls. The study also reported increased expression of COX-1 and COX-2, indicating that the enhanced PGE_2_ production was associated with elevated COX expression (Wang et al., 2003). This indicated that the magnitude of mechanical stretch influences the inflammatory response in tendon cells (Yang et al., 2005; Chen W. et al., 2015).

Mechanical loading significantly influences the expression of various neuropeptides (neuropeptide Y [NPY], Substance P, Calcitonin gene-related peptide [CGRP]) and neurotransmitters (Catecholamines) in tendons, affecting both their healing and pathological processes (Table 12). An augmented expression of NPY involved in various physiological processes, including pain modulation and delayed tissue remodeling could be observed after mechanical stimulation in tendons (Bjur et al., 2009; Chen et al., 2021). Another neuropeptide is Substance P, which is a mechanoinduced regulator of human Achilles tenocyte proliferation (10% strain with a frequency of 1 Hz for a total of 120 min) and creates therefore probably a healing effect (Backman et al., 2011).

**TABLE 12:** Effect of mechanostimulation on secretion of cytokines.

Protein	Affected cell population (cell density and adhesion time)	Culture system/device	Stretch parameter and duration	Observations	References
Cell response					
Cytokine					
IL-1β	Human tendon fibroblasts; (2–5 × 10^5^ cells·100 µL ^−1^·BAT^-1^)	3D bioartificial tendon (BAT) (Flexcell™ International)	Uniaxial strain 3.5%; 1 Hz; 1 h/d for 1, 3 and 5 days	IL-1 could promote cell survival under strain	Qi et al. (2011)
IL-1β	Human patellar tenocytes; (2 × 10^5^ cells in 6 × 3 cm silicon dish, 12–16 h)	2D *in vitro* model	4% or 8%; 0.5 Hz; 4 h	↓ IL-1β after 4%; ↑ IL-1β after 8%	Yang et al. (2005)
IL-6	Bovine common digital extensor tendon fascicles; (N.A.)	3D *ex vivo* model;	30%; 1 h	↑ IL-6	Legerlotz et al. (2013)
IL-6	Bovine common digital extensor tenocytes; (24 h)	3D *in vitro* scaffold model;	5%; 1 Hz; 24 h	↑ IL-6	Patel et al. (2018)
PGE2	Human patellar tenocytes; (2 × 10^5^ cells in 6 × 3 cm silicon dish, 12–16 h)	2D *in vitro* model	4% or 8%; 0.5 Hz; 4 h	↓ PGE2 after 4%; ↑ PGE2 after 8%	Yang et al. (2005)
PGE2	Human patellar tenocytes; (2 × 10^5^ cells, 36–48 h)	2D *in vitro* model	4%, 8%, or 12%; 0.5, 1, or 1.5 Hz; 24 h	8% and 12% magnitudes for 24 h significantly stimulated PGE2 release	Chen et al. (2015a)
Substance P	Human Achilles tenocytes; (1.75 × 10^5^ cells, 24 h)	2D *in vitro* model (Bioflex; BF-3001C)	10%; 1 Hz; 2 h/d; 3 days in sum	↑ Substance P	Backman et al. (2011)

Abbreviations: d, days; h, hours; Hz, Hertz; IL, interleukin; PGE2, Prostaglandin E2.

Nevertheless, the question arises as to how the mechanoresponse of tendon cells is influenced during healing processes.

## Mechanoimpulses and tendon healing response

The ability of tendons to regenerate is largely restricted by their hypocellularity, limited blood supply, low proliferation rate and the post-traumatic inflammation (Nichols et al., 2019). In principle, the healing process can be divided into four overlapping phases (hemorrhagic, inflammatory, proliferation, remodeling phases) (Schulze-Tanzil et al., 2022; Schneider et al., 2018). Directly after tendon injury, the **hemorrhagic phase** leads to bleeding and also to a release of platelets and immune cells, such as neutrophils, lymphocytes and other leukocytes. The latter initiate an inflammatory response (**inflammatory phase**) (Rosenbaum et al., 2010). No studies were found, that investigated the effect of mechanical loading on tendons during the hemorrhagic phase. The inflammatory phase is triggered in the first 72 h after an injury via autocrine and paracrine signals. During this inflammatory phase, macrophages migrate into the ruptured structure and the adjacent tissue and phagocytize tissue fragments and necrotizing material (Manning et al., 2014). Despite so far not investigated for tendon healing, macrophage`s proinflammatory M1 polarization was enhanced by 3% strain (Babaniamansour et al., 2024). Growth factors (e.g., platelet derived growth factor [PDGF], epidermal growth factor [EGF], IGF, vascular endothelial growth factor [VEGF] and TGF) are released, which promote granulation and neovascularization due to a chemotactic effect on endothelial cells and tenocytes (Molloy et al., 2003). This stimulation causes proliferation, synthesis of collagen types I, III, V and non-collagenous proteins and angiogenesis (**proliferation/granulation phase**). As a follow up to this process of new ECM synthesis, the orientation of the cells in longitudinal rows and the subsequent predominately parallel orientation of the collagen fibers play an important role in order to regain the structural and functional tissue features (**remodeling phase**). This phase is characterized by decreased cellularity, reduced collagen and glycosaminoglycan synthesis, and the transition of repair tissue into a reorganized functional tissue. The ECM and cell realignment represent processes strongly guided by mechanical strain. Introducing mechanical stimuli shortly after tendon injury can modulate the healing environment. However, excessive loading during the initial inflammatory phase may disrupt the delicate balance required for effective healing (Hammerman et al., 2024). Mechanical loading during the proliferative phase (5 days post-injury) of tendon healing has been shown to increase the quality and efficiency of rat Achilles tendon repair by increasing tendon-related gene expression (Eliasson et al., 2009). Specifically, loading increased the expression of inflammation-related genes such as IL-1β, inducible nitric oxide synthase (iNOS), prostaglandin E synthase (PGES), IL-6, and Chemokine CC motif ligand 7 (CCL7). These findings suggest that mechanical loading during the proliferation phase can modulate both inflammatory responses and tissue remodeling processes (Hammerman et al., 2018; Hammerman et al., 2017).

Mechanical loading during the **remodeling phase** of tendon healing (typically commencing around 4–6 weeks post-injury) is pivotal in enhancing tissue organization, strength, and function. Appropriate mechanical stimuli during this period can significantly influence the quality of tendon repair and overcome a frequent complication that is post-traumatic tendon heterotopic ossification (HO). Interestingly, certain tendon-specific factors, such as tenomodulin are involved in prevention of tendon HO. Delgado et al. have shown that loss of tenomodulin results in significantly increased HO during the tendon remodeling phase in the mouse Achilles tendon, which was concomitant with compromised biomechanical properties (Delgado Caceres et al., 2021). Mechanical loading facilitates the reorganization of collagen fibers, promoting their alignment along the direction of tensile forces. This alignment enhances the mechanical properties of the tendon, increasing its stiffness and tensile strength. Additionally, loading stimulates the formation of covalent bonds between collagen fibers, further strengthening the tissue. These adaptations contribute to the restoration of tendon function and reduce the risk of re-injury. Hence, appropriate mechanical stimuli are essential to promote healing, maintain tendon homeostasis and prevent the development of tendinopathy (Stanczak et al., 2024).

However, neither the morphological structure nor the biomechanical properties can be fully restored to their original state after a tendon rupture. This is due to the abnormal concentration, organization and cross-linking of collagen type I fibrils (crosslinks) (Frank et al., 1992; Frank et al., 1995). Furthermore, the ratio of collagen types I and III is unfavorable and even if there is an increase in collagen type III fibers during the initial repair phases, these are thinner than those in healthy tendons (Eriksen et al., 2002). The stability of repaired tendons is reduced by altered properties such as the degree of cross-linking, the fibril thickness, density and composite formations with other ECM components. The healed tissue is characterized by the variant composition of the tendon neo-ECM with other ratios of collagen and proteoglycan types. In addition to local molecular factors, differing from the non-injured tendon ECM, novel heterogeneous cell populations, either immigrated or amplified during healing could be detected (Freedman et al., 2018; Andarawis-Puri and Flatow, 2018; Maffulli et al., 2002; Chamberlain et al., 2011). Furthermore, the intimate interaction between resident (“intrinsic”) cell populations and extrinsic cells, including immune cells, might substantially change the overall mechanoresponse in a healing tendon, unless they decline over time.

Cells respond intimately to their biomechanical microenvironment, such as ECM stiffness. Therefore, this complex healing scenario gives rise to a multitude of questions. For example, how could the synchronized mechano-related cell-cell communication being reestablished in the damaged tendon during healing, or how to coordinate the mechanoresponse in a functional manner again? The posttraumatric inflammatory microenvironment might strongly influence cellular mechanoresponse but it still remains unclear how this microenvironment is exactly changed. An issue that demands further research.

Insufficient tendon healing has been recently considered as a result of a lack of vascularization, cell rounding, disorganization of the collagens in the ECM, neuronal ingrowth, persistence of inflammation and a change in metabolic processes (Wunderli et al., 2020). All these parameters might change the mechanical niche in healing tendons. It remains questionable whether and if so, how these unfavorable processes impact and lead to aberrant tendon healing and how could they be counteracted by a properly adapted exercise protocol. Since tendon healing partially recapitulates steps of tendon development, a more detailed understanding of the role of mechanobiology in developmental tenogenesis would be helpful in the future. Meanwhile, it is well known that mechanical stimulation is crucial in the early intrauterine development of tendons (Okech and Kuo, 2016). This observation supports the conclusion that mechanical stimuli have a significant influence on the tendon ECM synthesis and maturation, which is helpful to properly guide healing processes biomechanically in the future. Moreover, exercise protocols might also be adapted to individual healing scenarios leading to personalized mechanostimulation.

In summary, it has clearly been shown that mechanostimulation has an effect on the healing process of tendon and ligament tissues (Young et al., 1998; Awad et al., 2000; Juncosa et al., 2003). However, it is still unclear which parameters (amplitude, frequency, direction and duration) represent an optimal condition for functional healing. The influence of distinct protocols of mechanostimulation on -sensation, -transduction and -response (e.g., change in cellular function), still needs to be clarified, since the mechanical stimulus can be exerted on the cells via or as a combination of stretching, fluid flow, hydrostatic pressure or shear stress.

## The effect of mechostimulation in 2D culture

Diverse devices for 2D stimulation of tenocytes and TSPCs could be used to enhance the proliferation rate, metabolism and directional deposition of ECM components (Gögele et al., 2021a; Chernak Slane and Thelen, 2014; Xu P. et al., 2021; Passini et al., 2021; Rowson et al., 2018). Mechanical stimuli in 2D are one reason for cell proliferation, migration, tissue remodeling, and cellular processes including metabolism, gene transcription and cell differentiation (Papalamprou et al., 2023; Morita et al., 2019; Huang et al., 2023). During 2D mechanotransduction numerous mechanosensitive ion channels become activated, chromatin conformation changes and activation of transcription factors, as well as the release of various growth factors (TGF-β, VEGF, PDGF, bFGF) takes place (Subramanian et al., 2018; Petersen et al., 2004; Skutek et al., 2001). The transmission of mechanical signals, sensed at the plasmalemma, transduced via actin stress fibers to the nucleus causes chromatin decondensation and thus, promotes the expression of tenomodulin, whereby tenocyte migration was increased (Xu P. et al., 2021) (Figure 2). In 2D, it could already be shown that Ca^2+^ mediated mechanosensing occurring via cyclic stretch activated the calmodulin-dependent protein kinase kinase 2 (CaMKK2)/5′adenosine monophosphate-activated protein kinase (AMPK) signaling and the (FAK)-cytoskeleton reorganization, and thereby, helps to maintain tenocyte phenotype and homeostasis (Huang et al., 2022). In another study, the authors demonstrated that TSPCs, mechanostimulated in 2D, responded to complex changes in gene and protein expression of integrins, MMPs and ECM proteins that is mediated via well-known mechano-regulated kinases, namely, p38 and ERK1/2 (Popov et al., 2015). We also showed that at 14% stretch in 2D culture, anterior cruciate ligament-derived fibroblasts aligned against the direction of stretch, thereby altering their cytoskeleton by becoming even more spindle-shaped and slender than in the unstimulated control (Gögele et al., 2021a). This was confirmed in a recent study using rat Achilles tenocytes in the same *in vitro* model (Fleischmann et al., 2024). This phenomenon is contrary to the situation in 3D tissues, whereby there is a parallel cell alignment in the direction of axial extension. However, 2D stretch is limited by the fact that cells are not surrounded by their own ECM providing their natural mechanotopographical niche (Leal-Egana et al., 2020). Moreover, differentiated tenocytes devoid of their ECM usually dedifferentiate in a 2D environment that might also affect their mechanosensing and -transducing pathways. At this point, it should be mentioned that most 2D mechanostimulation experiments are carried out by means of cyclic stretching via stretched silicone membrane of different stiffness. There is a great need to study more thoroughly the effects of hydrostatic pressure or shear stress on tendon-relevant cell types.

## 3D stimulated cell-material constructs

3D stimulation of tendon-derived cells requires a 3D cell carrier to optimize tendon biofabrication applications. There are numerous biomaterials (e.g., diverse synthetic and natural polymers including collagen, silk forming scaffolds (Shiroud Heidari et al., 2023; Xue et al., 2022) or hydrogels (Wan et al., 2024; Bs et al., 2024; Li S. et al., 2023) that are used in the tendon biofabrication field based on the fact that they try to mimic the native tendon ECM as closely as possible (Gehwolf et al., 2019; Stoll et al., 2011; Hahn et al., 2019). These scaffolds alone, already have some capacity to guide regeneration due to similar mechanical strength compared to the native tendon/ligament (Hahn et al., 2019; Miescher et al., 2024). It has been shown that precolonized scaffolds with primary human adipose-derived mesenchymal stem cells (hADMSCs) or induced pluripotent stem cell-derived mesenchymal stem cells (iPSCs) can support defect healing *in vivo* even more efficiently compared to uncolonized scaffolds (Jiang et al., 2020; Kaneda et al., 2023). The interplay between scaffold, cells and mechanical loading is not yet fully understood. The phenotypical switch of macrophages towards M2 that is potentially beneficial for tendon healing, should be investigated in more detail in the future (Schoenenberger et al., 2020). Bioreactors are required to cultivate cell-loaded scaffolds *in vitro* in order to be able to properly simulate the natural microenvironment (Gögele et al., 2021b). The use of force-transmitting 3D bioreactors allows us to understand the mechanobiology in more detail, thereby reducing the number of *in vivo* experiments. Like in 2D, ECM synthesis and cell differentiation are supported by cyclic stretch (Burk et al., 2016; Zhang et al., 2018). In contrast to 2D, cells start alignment parallel to the direction of stretch, very similar to their orientation in native tendon. However, similar to 2D culture, there is still no uniform consensus on the duration, amplitude and frequency of stimulation under 3D conditions to promote tenogenesis by using different tenocyte subpopulations or stem cells (TSPCs or mesenchymal stromal cells [MSCs] from bone marrow or fat tissue) (Chen et al., 2022; Wang et al., 2018b; Rinoldi et al., 2019; Russo et al., 2022; Xu et al., 2015). In most 3D studies, stimulation is exerted by cyclic stretch. Fluid flow and pressure are also expected to stimulate tenogenic cells, but should be validated more extensively in the coming years. Moreover, the stimulation protocols must be properly adapted to the respective cell types. However, for this reason, the comparability of the different studies on the tendon mechanotransduction under 3D conditions is limited and the interpretation of results remains controversial, due to the specific advantages and disadvantages of different systems.

## Advantages and disadvantages of different 2D and 3D stretch devices

Depending upon the research question addressed, there is now a whole range of different devices for 2D and 3D stimulation of tendons or tenogenically differentiated cells. A brief overview of the advantages and disadvantages of 2D (Table 13) and 3D (Table 14) stimulators is given.

**TABLE 13:** The advantages and disadvantages of different 2D stretch devices for tenocyte/tendon stimulation.

2D stretch devices	Description	Advantages	Disadvantages
Pneumatic stretching devices	These devices use pneumatic actuators to deform an elastic membrane on which cells are cultured, applying cyclic or static strain	Capable of applying uniform and controlled strain across the cell culture surface. Can simulate physiological stretching conditions by adjusting pressure parameters. Suitable for live-cell imaging during stretching (Constantinou and Bastounis, 2023)	Requires precise control systems to maintain consistent pressure and strain. Potential for mechanical wear and tear of the membrane over time
Electromagnetic stretching devices (Kamble et al., 2017)	Utilize electromagnetic actuators to apply uniaxial or biaxial strain to cells cultured on elastic membranes	Precise control over strain parameters, including magnitude and frequency. Rapid response times suitable for dynamic stretching protocols	Potential heating effects from electromagnetic components may affect cell viability. Complex setup, requiring integration of electromagnetic systems with cell culture environments
Motor-driven stretching devices (Riehl et al., 2012; Gögele et al., 2021a; Kubo et al., 2020; Skutek et al., 2001)	Use stepper or servo motors to mechanically stretch elastic membranes or scaffolds on which cells are cultured	High precision in controlling stretch parameters. Capable of applying complex strain patterns, including uniaxial and biaxial stretches. Simple design and relatively easy to implement. Effective for applying consistent uniaxial strain	Mechanical components may introduce vibrations that can affect cell behavior. Generally larger and more complex, requiring more space and maintenance. Limited to uniaxial stretching; not suitable for biaxial or complex strain patterns. Potential for edge effects where clamps grip the substrate, leading to non-uniform strain distribution

**TABLE 14:** The advantages and disadvantages of different 3D stretch devices for tenocyte/tendon stimulation.

3D stretch devices	Description	Advantages	Disadvantages
Motor-driven stretching devices, Scaffold Fixation mechanism	Clamps or grips that securely hold the tendon scaffold at both ends (Burk et al., 2016; Gögele et al., 2021b)	- **Promotion of Cell Proliferation:** Appropriate mechanical loading can enhance the proliferation of tendon-derived stem cells, contributing to tissue regeneration (Xu et al., 2015) - **Enhanced Tenogenic Differentiation:** Cyclic stretching in a 3D environment has been shown to upregulate tendon-specific markers, such as scleraxis and type I collagen, indicating successful differentiation of stem cells into tendon-like cells (Riehl et al., 2012); increase cell viability and synergistically promote the differentiation of BMSCs into tenocytes (Zhang et al., 2018); promote total collagen secretion (Wu et al., 2017a) - **Improved ECM Organization:** Mechanical stimulation promotes the alignment of collagen fibers and cells along the direction of strain, resulting in a more organized and functionally relevant ECM structure (Riehl et al., 2012; Wu et al., 2017b; Wu et al., 2016) - **Physiological Relevance:** Compared to two-dimensional systems, 3D cyclic stretch devices mimic more accurately the native mechanical environment of tendons, leading to more physiologically relevant cellular responses (Sheng et al., 2020) -	- **Technical Complexity:** Designing and operating 3D cyclic stretch bioreactors require specialized equipment and expertise, which can be a barrier for some research settings (Burk et al., 2016) - **Standardization Challenges:** Variability in scaffold materials, cell types, commercially available standardized devices and mechanical loading parameters can lead to inconsistent results, making it difficult to standardize protocols across different studies - **Limited Nutrient Diffusion:** In thicker 3D constructs, ensuring uniform nutrient and oxygen diffusion throughout the scaffold can be challenging, potentially affecting cell viability in the core regions - **Scalability Issues:** Transitioning from laboratory-scale models to clinically relevant sizes remains a significant hurdle, particularly in maintaining uniform mechanical stimulation and cell distribution

BMSC, bone marrow derived mesenchymal stem cells; ECM, extracellular matrix.

It should be emphasized at this point that stimulation devices are still needed that can simulate all types of mechanical stimuli in the tendon. With the help of these devices, it may be possible to establish tendon defects/implantation models on chip, in which human cells are also used, so that a regenerative approach can be developed. Another point that has not yet been addressed and should be investigated further in the future, is the fact that tendons and ligaments are usually not stretched in just one direction. For example, in the anterior cruciate ligament, there is both translational and rotational movement of the tissue. To date, there are no commercial devices that can better depict both movement sequences and thus the native state.

## Discussion

To summarize, it can be concluded that moderate cyclic mechanical stimuli lead at the cellular level to enhanced cell-ECM interactions and the activation of anabolic molecular processes that are important for tissue homeostasis and supportive for tendon healing. To deepen the understanding of tendon mechanobiology new methods such as RNASeq (transcriptomics) are currently being used to distinguish by single cell analysis multiple novel cell subpopulations in the tendon that react and possibly interact in a diverse manner in response to different mechanical challenges (Marr et al., 2021; De Micheli et al., 2020; Sarmiento and Little, 2021; Ramos-Mucci et al., 2022; Kendal et al., 2020).

The tendon-on-chip technique would be an interesting tool for tissue or single cell analyses (Bakht et al., 2024). The advantage of tendon-on-chip technology compared to others is that a mechanoniche with several cell types could be created in a very small space. It allows us to gain a better understanding of healthy tissue and the causes of tendon fibrosis by investigating the intimate crosstalk between tenocytes and distinct inflammatory mediators (Awad et al., 2025; Ajalik et al., 2025). In order to better characterize the native conditions *in vitro*, organ-on-chip techniques with additional fluid flow were designed to mimic vascular microenvironments of injured tendons (Linares et al., 2025). Crosstalk analysis between different cell types under biophysiological and biochemical conditions has also been tested with human-adipose tissue derived stem cells to study tendon physiology (Monteiro et al., 2023). The organ-on-chip technique can be used not only for tendons but also to gain research insights into chronic inflammation of the enthesis (Giacomini et al., 2024). Although, it should be added that it has so far only been tested with fluid flow and has not been subjected to dynamic cyclic stretch conditions, which would be very helpful for the future and would allow us to stimulate extensive mechanical stress or overload that can also induce tendon pathologies. In addition, tendons compromised by background diseases (e.g., diabetes) or aging might respond in a different manner to mechanical challenges, and this could be investigated in tendon-on-chip disease models.

Age-related changes in tendon mechanosensitivity involve alterations at cellular, structural and functional levels, impacting the tendon’s ability to respond and withstand to mechanical stimuli (Grote et al., 2019; Thampatty and Wang, 2018). Aged tendons exhibit changes in mechanotransduction pathways, leading to diminished responses to mechanical loading (Aggouras et al., 2024; Dudhia et al., 2007). This includes alterations in focal adhesion components and cytoskeletal organization that are critical for mechanosensing (Arnesen and Lawson, 2006; Kwan et al., 2023; Leahy et al., 2024).

Such changes can impair the tendon’s adaptive responses to mechanical stress (Epro et al., 2017; McCarthy and Hannafin, 2014; Kwan et al., 2023). Furthermore, aging decreased cellularity in tendons and tenocyte`s proliferation and metabolic activity. This decline in cellular functions affected the tendon’s capacity for repair and adaptation to mechanical stimuli (Lui and Wong, 2019; Svensson et al., 2016).

Tenocyte senescence in aged tendons impairs the capacity for ECM remodelling (Stowe et al., 2024). Hence with aging, tendons undergo structural modifications, such as disorganized collagen fiber arrangement and increased presence of non-tendinous tissue components, such as fatty deposits and calcifications. These changes can compromise the tendon mechanical integrity and its ability to transmit forces effectively to fulfil its key function (Lui and Wong, 2019; Goh et al., 2018).

The discussion of age-related changes in mechanosensitivity can only be dealt with briefly here, since as there are now many comprehensive studies on this topic (Epro et al., 2017; Korcari et al., 2023; Carroll et al., 2008). The study by Aggouras et al. (2024) confirmed that young and aged tendons display different mechanisms of ECM remodeling in response to tensile loading, supporting the notion that tendon pathologies require age-specific clinical interventions and therapies (Aggouras et al., 2024). In addition, Kwan and colleagues (2023) have already published a review on this topic and no more recent literature could be found that investigated the difference in the effects comparing 2D and 3D mechanostimulation of aged tenocytes *in vitro* or *in vivo* (Kwan et al., 2023).

A better understanding of mechanotransduction is also needed, in order to treat tendon injuries with the help of pre-stimulated cell-seeded 3D scaffolds. Transferring mechanobiological principles into the design of biomaterial scaffolds is pivotal for enhancing tissue regeneration by amplified cell proliferation and ECM synthesis. Mechanomimetic scaffolds can be engineered to optimally transduce mechanical cues that guide tissue formation. The key strategies for designing these scaffolds are to provide an optimized scaffold architecture (e.g., by adapting porosity, pore size, fiber alignment), avoiding the risk of cell squeezing under strain, the utilization of mechanically instructive and sufficiently resilient materials (with appropriate stiffness, elasticity and surface topology) (Zhang et al., 2012; Li et al., 2024; Huang et al., 2023).

Another important aspect for studying tendon mechanobiology *in vivo* is to take different species (horses, cow, pigs, sheep, rabbits, dogs, rats and mice) in account with respect to animal models with significant importance for translational applications. These differences manifest in tendon structure, cellularity, cellular composition, mechanical properties and mechanoresponses. Therefore, it is important to choose the appropriate animal model, closest reflecting human conditions and species to harvest tenocytes for tendon research (Burgio et al., 2022; Oreff et al., 2021).

A comprehensive review highlights that many animal models, particularly rodents, do not fully replicate human tendon anatomy and loading conditions (Hast et al., 2014; Aykora et al., 2025). Mechanical loading induced different cellular and molecular responses in animal compared to human tendon. Cyclic loading in rodent models may lead to different adaptations than observed in human tendons (Stanczak et al., 2024; Magnusson and Kjaer, 2019). Quadrupedal animals distribute mechanical loads differently than bipedal humans, affecting tendon adaptation and therefore, the healing process (O'Neill et al., 2022; Roberts et al., 1998). In this context, it should also be mentioned that, depending upon topography and type, not all tendons are the same in their mechanoresponse, even in the same species.

In our view, no 2D or 3D stretch protocol investigated *in vitro* can yet be directly transferred to *in vivo* conditions due to complexity of the *in vivo* mechanical environment (Wang et al., 2018a), the differences in mechanical tendon properties (Bossuyt et al., 2024), the variations in cellular responses, the limitations in replicating physiological conditions (Escriche-Escuder et al., 2022; Stirling and McEachan, 2023) and the challenges in measuring and applying appropriate loads. In other words, a large-scale study would be needed to demonstrate direct transferability. An interdisciplinary team has also been recommended to work in multidisciplinary collaboration of doctors, scientists and therapists to use primary patient cells for the 2D and 3D stretcher and to establish a treatment protocol for patients in parallel (Wang et al., 2018a). Furthermore, this should be carried out with humans and not with animal models, as there is only limited direct transferability. Due to the different localizations (Achilles tendon, rotator cuff tendons, cruciate ligament), a direct transfer from an *in vitro* stretching protocol to the rehabilitation protocol in humans cannot be carried out, so the authors did not want to make any statement within the present review. The current treatment protocols for patients after Achilles tendon ruptures are also very different, depending on whether surgery was performed or not and especially in terms of timing weight bearing, immobilization strategies and rehab milestones (Saxena et al., 2022; Cohen et al., 2023).

## Further perspectives for targeted tendon repair by mechanical loading

Looking at the mechanomics of each tendon cell population promises a great potential for future discoveries into the mechanobiology of tendons and mechano-induced regeneration approaches. In addition, the large number of different stretching protocols, the wide range of different stretching devices and the high costs of 3D bioreactors mean that standardized parameters for biofabrication are still in demand. Further developments in the field of tendon reconstruction in the future will include 4D applications and direct *in situ* bioprinting using robotics (Patrawalla et al., 2024).

## Conclusion

Tenocyte subpopulations, TSPCs, myofibroblasts, and MSCs provide a cellular basis for tendon tissue engineering due to their mechanosensitivity. Meanwhile, numerous 2D and 3D *in vitro* and *in vivo* studies have shown that mechanical stimulation is primarily necessary to maintain tendon homeostasis and to stimulate tissue formation. However, there are still challenges in translating findings from mechanical stimulation to clinical applications. Mechanocompetent and tenoconductive 3D biomaterials serving as a cell carrier with sufficient cell adhesion motifs could be used in conjunction with appropriate mechanical stimulation of these cells, as an implant to treat tendon defects. These tissue engineered constructs could accelerate tendon regeneration in the future. To achieve this vision, first, uniform and sophisticated 3D models are required, reflecting the complex loading conditions (cyclic stretch, pressure, through fiber torsion, fluid flow and shear forces) of the particular tendon being investigated. These models should also rebuild the 3D mechanotopographical niche of the respective investigated tendon with its resident cell populations. These tools will be helpful to elucidate the mechanomics of tendons in more detail. Nowadays, the emerging tendon on chip platforms have started to address many of these requirements. Furthermore, identification of pharmaceutically addressable mechanosensitive ion channels or receptors, as a target to accelerate tendon regeneration is needed. Altogether, such cumulative efforts could lead to major steps towards more efficient and satisfactory management of tendon pathologies.
